# Associating local strains to global pressure–volume mouse lung mechanics using digital image correlation

**DOI:** 10.14814/phy2.15466

**Published:** 2022-10-07

**Authors:** Talyah M. Nelson, Kathrine A. M. Quiros, Crystal A. Mariano, Samaneh Sattari, Arzu Ulu, Edward C. Dominguez, Tara M. Nordgren, Mona Eskandari

**Affiliations:** ^1^ Department of Mechanical Engineering University of California Riverside California USA; ^2^ BREATHE Center School of Medicine University of California Riverside California USA; ^3^ Division of Biomedical Sciences School of Medicine, University of California Riverside California USA; ^4^ Department of Bioengineering University of California Riverside California USA

**Keywords:** compliance, digital image correlation, heterogeneity, lung, real‐time, strain

## Abstract

Pulmonary diseases alter lung mechanical properties, can cause loss of function, and necessitate use of mechanical ventilation, which can be detrimental. Investigations of lung tissue (local) scale mechanical properties are sparse compared to that of the whole organ (global) level, despite connections between regional strain injury and ventilation. We examine ex vivo mouse lung mechanics by investigating strain values, local compliance, tissue surface heterogeneity, and strain evolutionary behavior for various inflation rates and volumes. A custom electromechanical, pressure–volume ventilator is coupled with digital image correlation to measure regional lung strains and associate local to global mechanics by analyzing novel pressure–strain evolutionary measures. Mean strains at 5 breaths per minute (BPM) for applied volumes of 0.3, 0.5, and 0.7 ml are 5.0, 7.8, and 11.3%, respectively, and 4.7, 8.8, and 12.2% for 20 BPM. Similarly, maximum strains among all rate and volume combinations range 10.7%–22.4%. Strain values (mean, range, mode, and maximum) at peak inflation often exhibit significant volume dependencies. Additionally, select evolutionary behavior (e.g., local lung compliance quantification) and tissue heterogeneity show significant volume dependence. Rate dependencies are generally found to be insignificant; however, strain values and surface lobe heterogeneity tend to increase with increasing rates. By quantifying strain evolutionary behavior in relation to pressure–volume measures, we associate time‐continuous local to global mouse lung mechanics for the first time and further examine the role of volume and rate dependency. The interplay of multiscale deformations evaluated in this work can offer insights for clinical applications, such as ventilator‐induced lung injury.

## Graphical abstract

Topological strain contours for representative mouse at various inflation volumes and frequencies

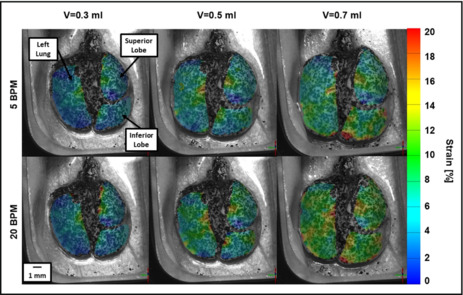

## 1 INTRODUCTION

Lung disease ranks as one of the top contributors of illness and mortality; chronic obstructive pulmonary disease (COPD) alone is one of the world's leading causes of mortality, joined by COVID‐19 in 2020 (Guarascio et al., 2013; Rattue, 2012; Stokes et al., 2020). Pulmonary disease alters tissue mechanical properties, which can be detrimental and lead to the irreversible loss of lung function (Eskandari et al., 2015; Eskandari et al., 2016); the resulting difficulty associated with spontaneous breathing necessitates interventions, such as mechanical ventilation. However, mechanical ventilation may also contribute to lung damage (Novak et al., 2021; Slutsky, 1993), and a key indicator of injury arising from mechanical ventilation is strain of the parenchyma (Paula et al., 2016). Mechanical strain is directly correlated with lung inflammation and damage (Retamal et al., 2018), and ventilator‐induced lung injury (VILI) is associated with elevated and imbalanced levels of regional lung tissue strain (Cruces et al., 2020; Eskandari et al., 2021; Kaczka, 2021). Furthermore, strain is thought to be exacerbated with use of high volumes and fast rates (Auten et al., 2001; Herrmann et al., 2018; Kaczka et al., 2015). Thus, examining local lung mechanical strain under various ventilation modes is important to improving our understanding of appropriate lung loading (Mariano et al., 2022).

Historically, the pressure–volume (PV) curve has offered critical advancements in characterizing lung mechanical behavior during ventilation (Harris, 2005; Jonson & Svantesson, 1999; Mitzner, 2011). However, regional lung tissue strains cannot directly be investigated from the bulk, organ‐level, global insights offered by the PV curve, which is needed to examine how altered organ‐level ventilation schemes impact local‐level lung damage. Previous studies have made fundamental strides in quantifying deformations, using methods such as bulk lung measurements as a surrogate for local strain (Chiumello et al., 2008), and microcomputed tomography (micro‐CT) scans (Hurtado et al., 2020) and digital volume correlation (DVC) (Arora et al., 2021). While these methods offer important insights for understanding regional tissue strains, these techniques may give rise to inaccuracies, and measurements are acquired at only select discrete time points and are projected and extrapolated. On the other hand, digital image correlation (DIC) offers high‐speed, noncontact, full‐field deformations of biological tissues in real time (Eskandari et al., 2022; Palanca et al., 2016; Rizzuto et al., 2016). Albeit limited to the surface of the organ, DIC can be used to directly characterize the regional distribution of strains as they evolve throughout the motion of a breathing cycle, instead of the equilibrated discrete snapshots. Thus, DIC can further account for pulmonary viscoelastic behaviors, which is central to assessing how various ventilation frequencies impact tissue strains during mechanical ventilation (Kaczka et al., 2015; Mariano et al., 2020).

Harnessing DIC to interface with a custom‐designed electromechanical ventilation device, we quantitatively and qualitatively characterize the local strain as it relates to global measures for mice lung specimens (Mariano et al., 2020; Sattari et al., 2020). Positive‐pressure inflation tests are executed under various volumes and breathing rates to quantify the evolution of local lung strain (i.e., the temporal change in strain and its development over the loading cycle) as it relates to the global PV inputs. The resulting associative local–global murine lung mechanical behavior is evaluated and, for the first time, is assessed over the entirety of the inflation cycle to characterize real‐time, continuous organ deformation heterogeneities and local compliances. The fundamental insights yielded by this novel study can help to improve our basic science understanding of lung mechanical function during physiological breathing and clinically, for lungs undergoing artificial ventilation.

## 2 MATERIALS AND METHODS

### 2.1 Sample preparation

**FIGURE 1:**
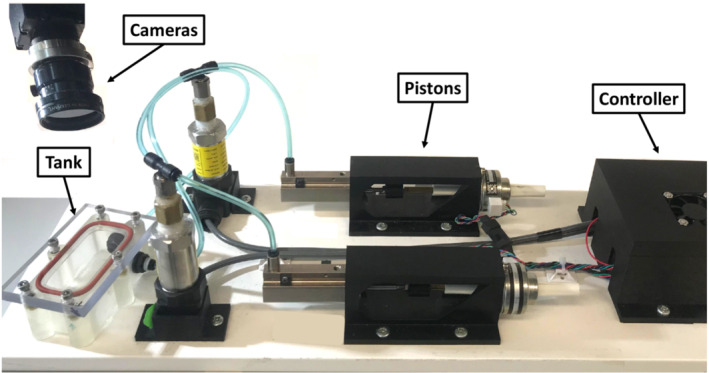
Testing configuration. The pressure‐measured, volume‐controlled system's tank sits below two DIC cameras, allowing the specimen's deformation to be recorded in three dimensions during inflation to assess the topological strain data.

Inflation tests were performed on ex vivo pulmonary specimens from five C57BL/6J mice subjects (31.3 ± 4.5 g), obtained from Jackson Laboratory at ages 8–12 weeks (Burr et al., 2021), and all procedures were approved by the University of California, Riverside (UCR) Institutional Animal Care and Use Committee (AUP#20200014). Mice were kept in microisolator cages at the UCR animal vivarium, and food and water were provided with unlimited access. These mice were part of an expanded study and served as the healthy control group intranasally exposed to 1X phosphate‐buffered saline (PBS). After 21 weeks, mice were euthanized and upon sacrifice, the lung was removed from the chest cavity and a cannula was inserted into the trachea. Using a syringe, the lung was inflated with 0.5 ml of air to avoid collapse during dissection. To keep the tissue hydrated, the lung was suspended in 1X PBS solution until testing commenced no later than 3 h postmortem. Lungs were ventilated via our custom‐designed volume‐controlled inflation–deflation system (Figure 1), while simultaneously collecting DIC data to assess the local strains.

To create the randomized speckling pattern on the specimen required for DIC, the sample was inflated to 0.5 ml and then airbrushed (Infinity CR 2 Airbrush, Harder & Steenbeck) with a hybrid water‐resistant white paint (ProAiir Makeup), until a thin layer of white paint coated the lung for sufficient imaging contrast as previously established (Mariano et al., 2020). A spray bottle was used to speckle the surface of the specimen with black hybrid water‐resistant paint to create small, randomized speckles for DIC displacement tracking.

### 2.2 Testing, data collection, and analysis

Once the lung was speckled, it was placed in the tank of our novel volume‐controlled, pressure measured, dual‐piston ventilation system that accounts for air compressibility in real time (Sattari et al., 2020). Inside the tank, the specimen was connected via tubing to a piston that inflated and deflated the lung by pushing air in and out. As previously detailed, a Trilion ARAMIS Adjustable 12 M dual‐camera imaging system, with a pixel resolution of 4096 × 3000, was directed at the specimen and used to collect DIC data (Mariano et al., 2020) (Trilion Quality Systems).

For the inflation tests, a preload of 5 cm H_2_O was used to create a matched datum state across tests and specimens (Sattari et al., 2020), with three subsequent inflation–deflation preconditioning cycles at the specified test volume and rate, followed by an analyzed fourth test inflation cycle. Comparable to those of previous studies, peak applied test volumes of 0.3, 0.5, and 0.7 ml at breathing rates of 5 and 20 breaths per minute (BPM) were used (Limjunyawong et al., 2015), and DIC was collected at 5 and 20 Hz for 5 and 20 BPM, respectively. These breathing rates (below 20 cycles/min) permitted testing within the quasi‐static realm, as time‐dependent behaviors (e.g., hysteresis) are shown to be predominantly due to the tissue structure, rather than flow effects, at low cycling frequencies (Bayliss & Robertson, 1939; Mount, 1955). Additionally, weight‐normalized tidal volumes are reported as 9.8 ± 1.3, 16.3 ± 2.2, and 22.8 ± 3.1 ml/kg, for the applied volumes of 0.3, 0.5, and 0.7 ml, respectively.

Analysis was conducted with the Trilion ARAMIS GOM software (Trilion Quality Systems GOM ARAMIS, 2016) using a facet size of 20 and a point distance of 15 pixels (Mariano et al., 2022). For details regarding the settings as well as the working principles of DIC and its application to lung ventilation, the authors direct the reader to previous work and the commercial software technical manual, which established use of DIC in a pilot murine lung study (Mariano et al., 2020; Trilion Quality Systems GOM ARAMIS, 2016).

**FIGURE 2:**
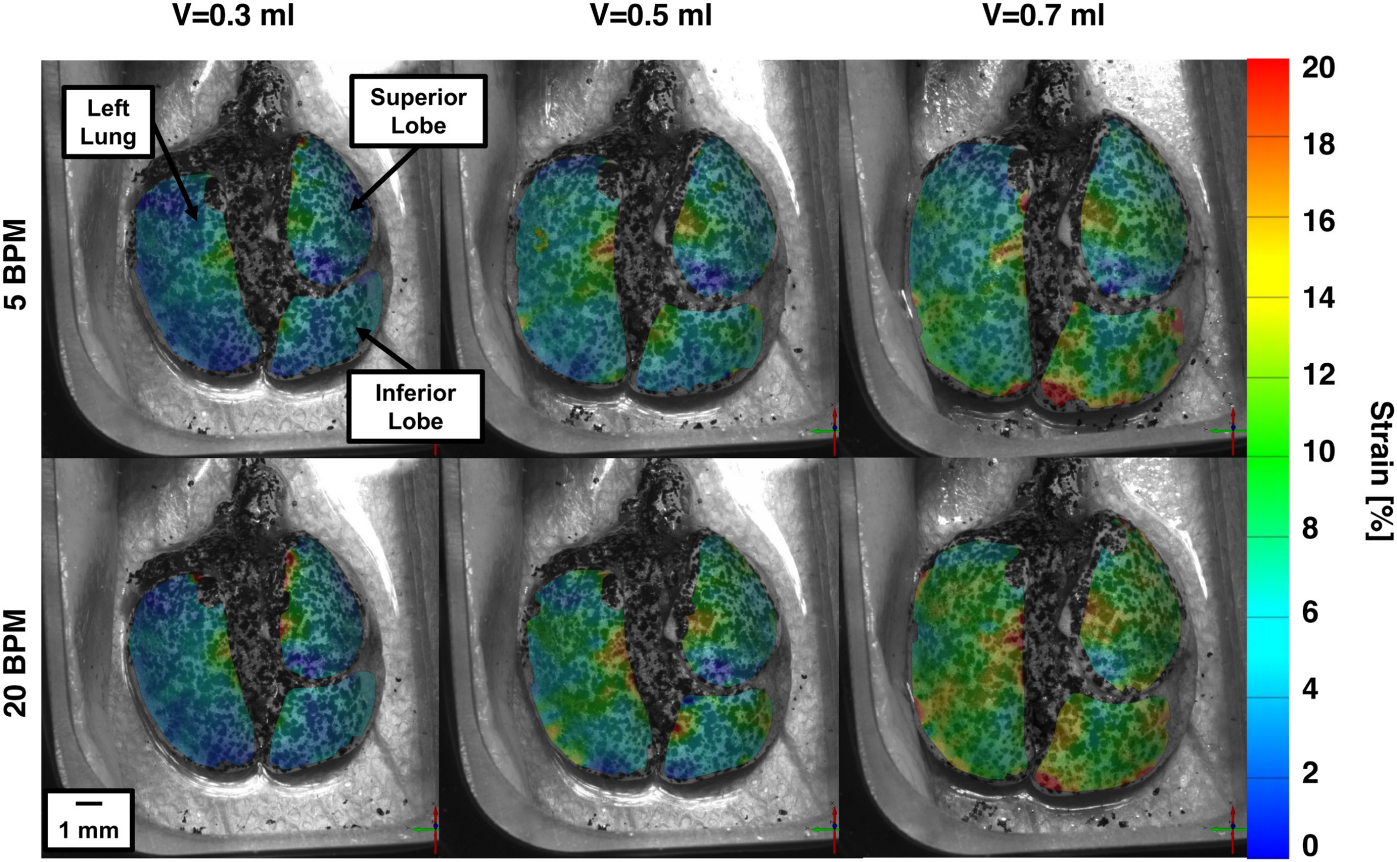
Topological strain contours at peak inflation for a representative mouse at various applied volumes (0.3, 0.5, 0.7 ml) and frequencies (5 and 20 BPM).

**FIGURE 3:**
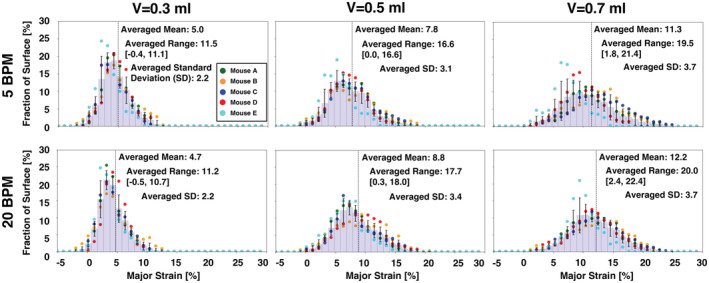
Histograms representing strain data obtained from DIC, averaged across all five mice specimens, at each peak inflation volume and breathing frequency. Strains are represented by the fraction of the lung surface on which they occur.

**FIGURE 4:**
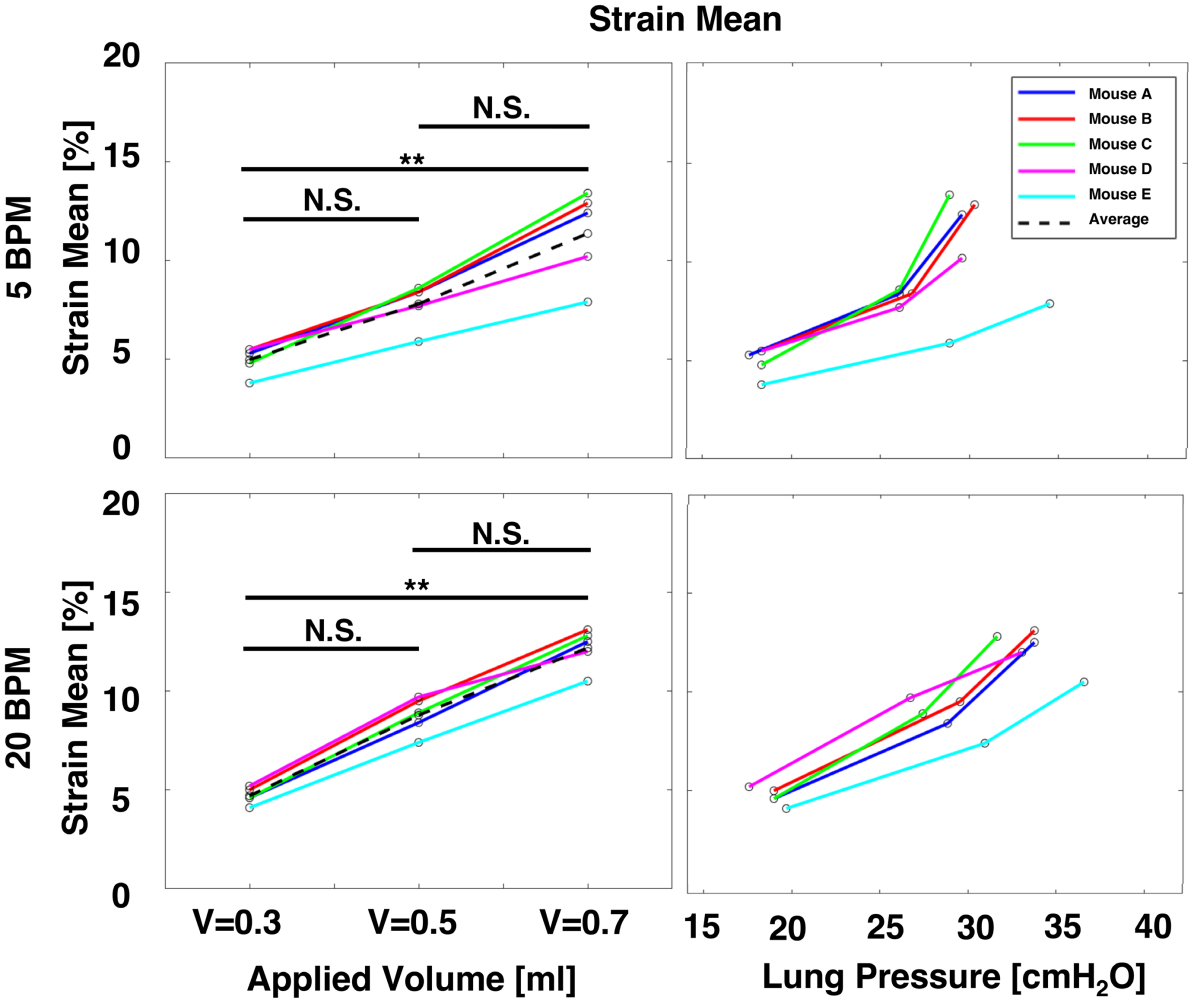
Specimens' strain mean at each applied volume and measured peak inflation is depicted as a function of increasing volumes and pressures.

**FIGURE 5:**
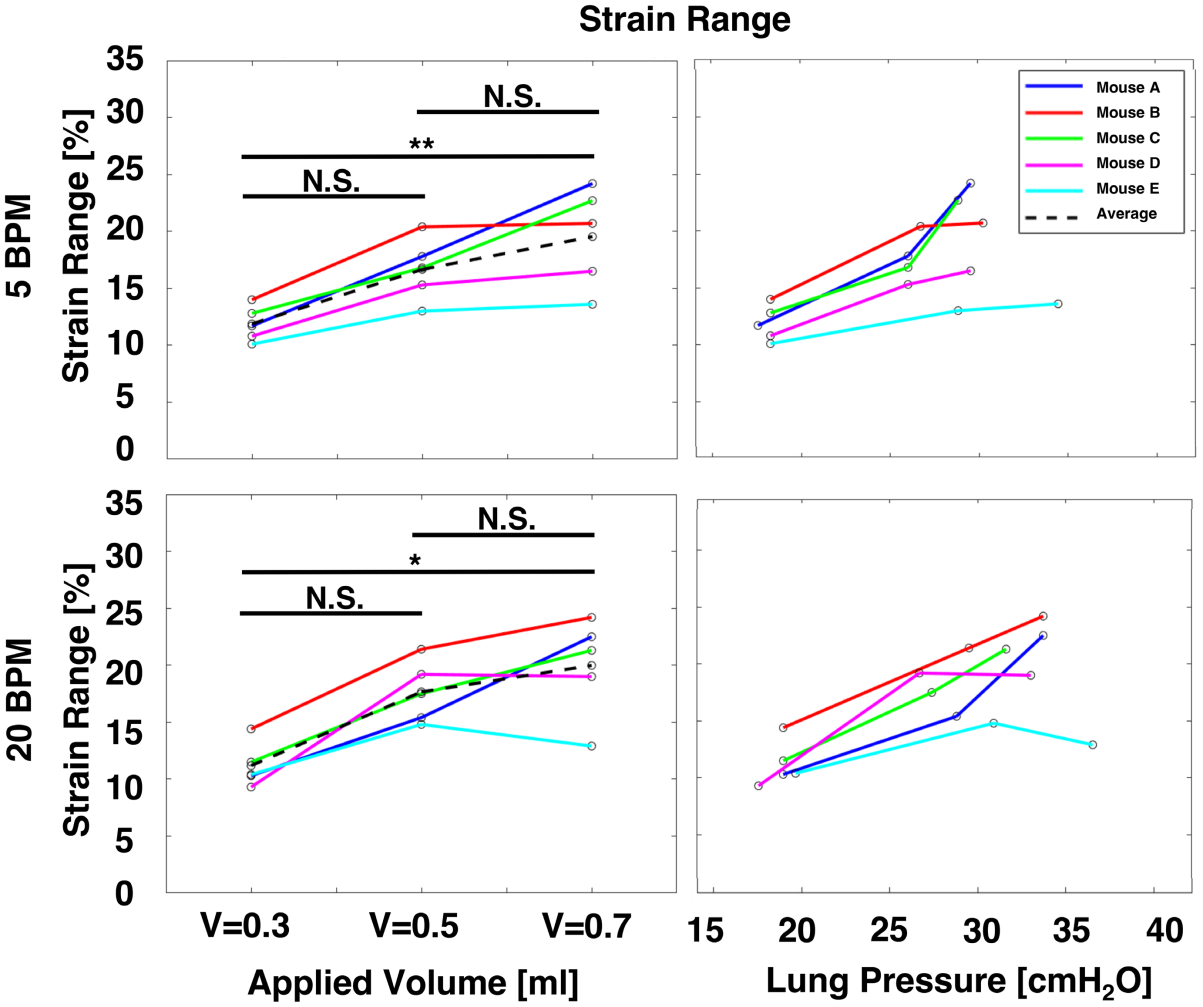
Specimens' strain range at each applied volume and measured peak inflation, for increasing volumes and pressures.

**FIGURE 6:**
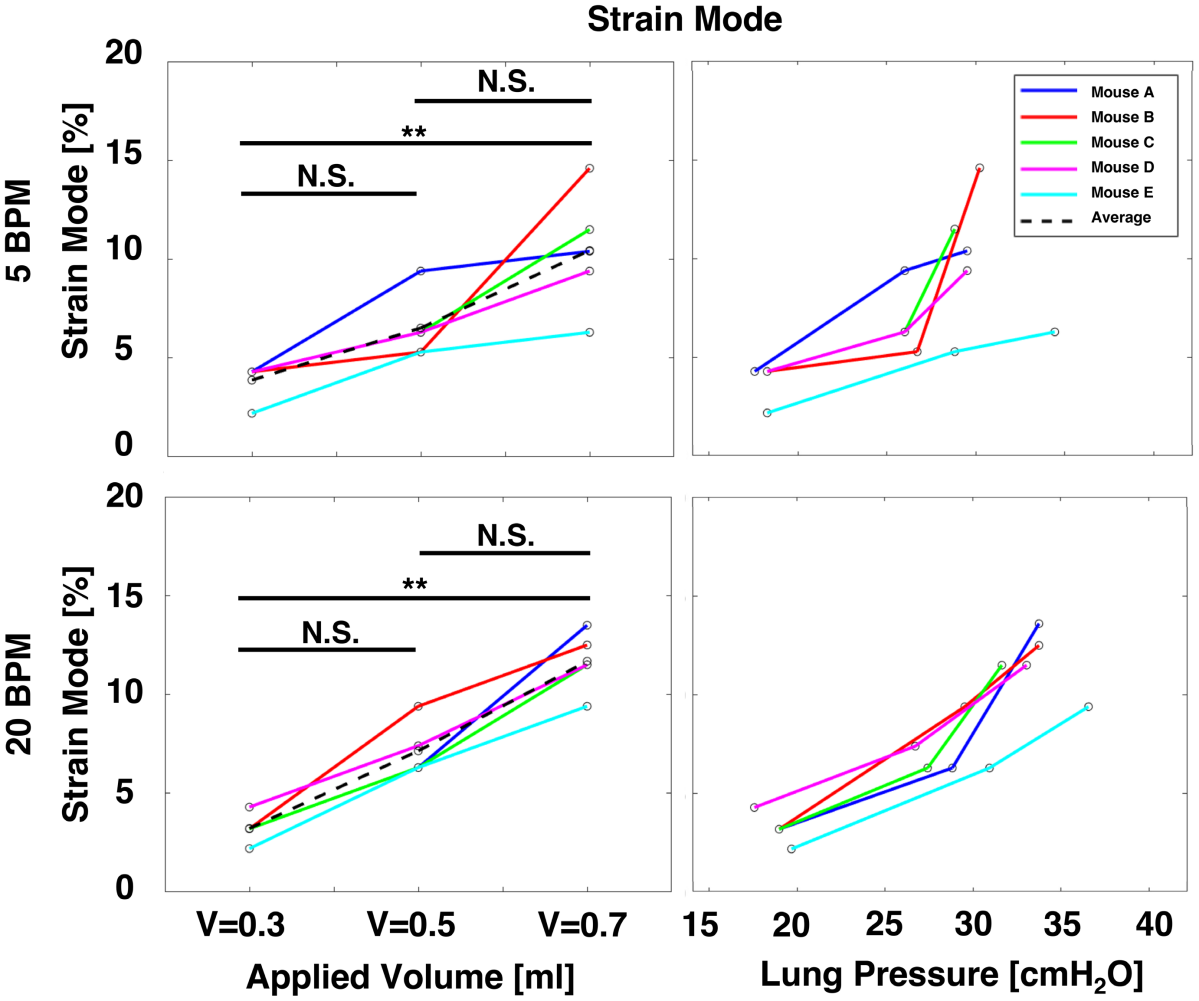
Specimens' strain mode at each applied volume and measured peak inflation, for increasing volumes and pressures.

**FIGURE 7:**
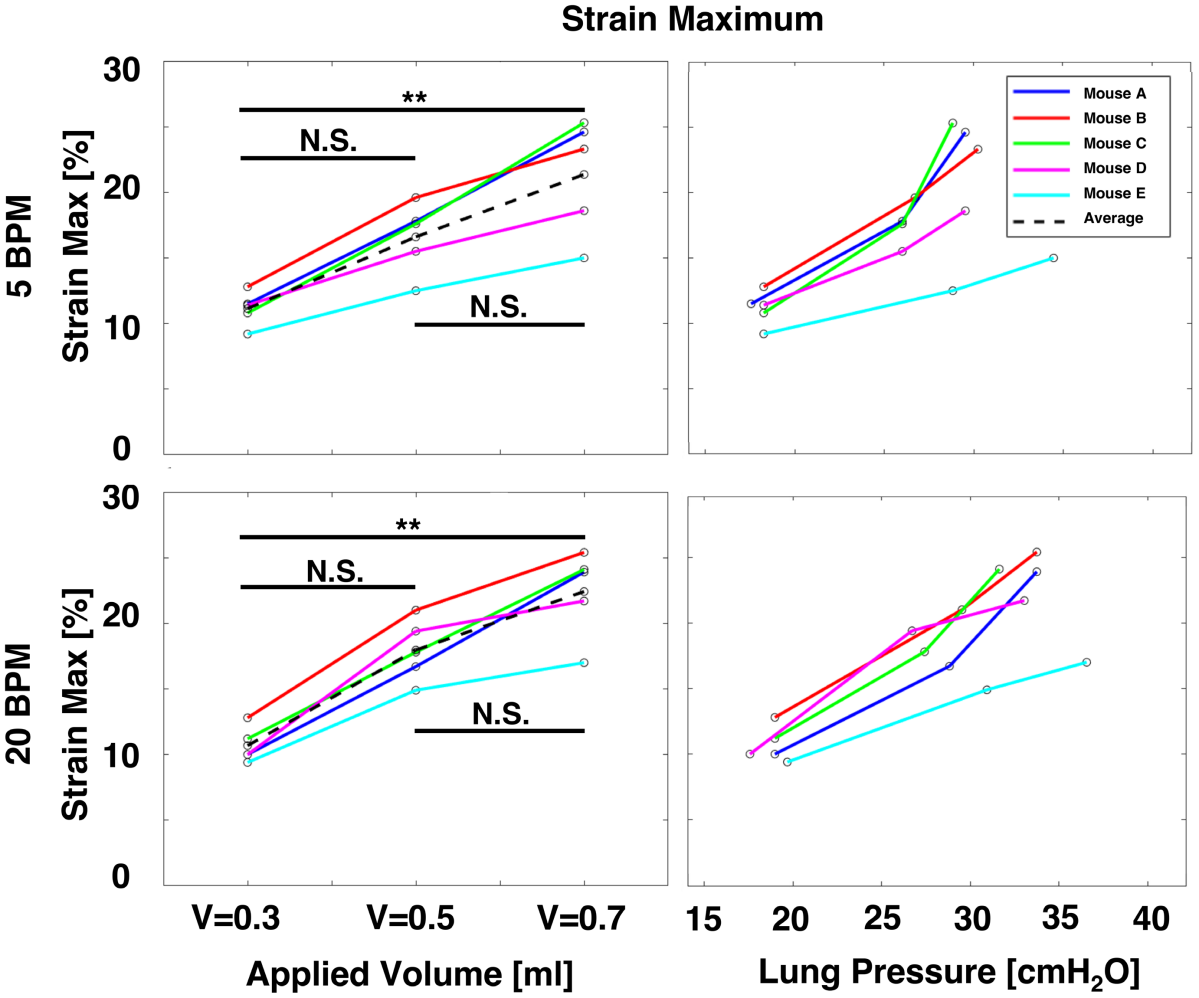
Specimens' strain maximum at each controlled applied volume and measured peak inflation shown as a function of increasing volume and pressure.

**FIGURE 8:**
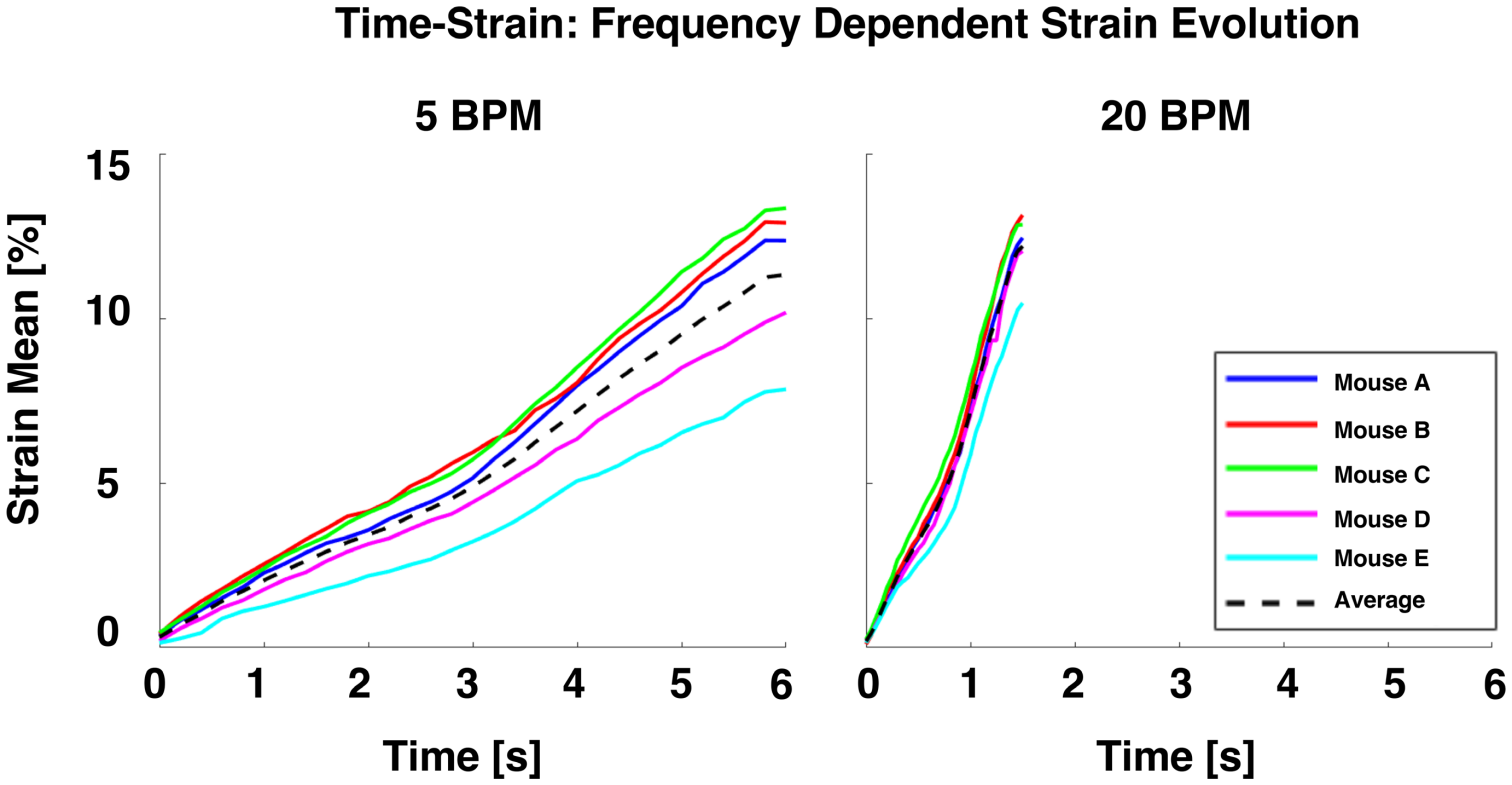
Specimens' individual and averaged temporal evolution of strain means for an applied (peak) inflation volume of 0.7 ml shown at the two breathing frequencies (5 and 20 BPM).

**FIGURE 9:**
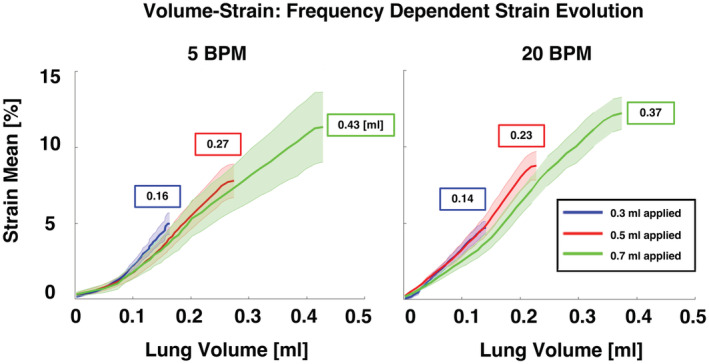
Specimens' volume evolution of strain means for each applied inflation volume of 0.3, 0.5, and 0.7 ml at two breathing frequencies (5 and 20 BPM). Curves represent the averaged strain mean and standard deviations across murine specimens. Show in the boxes are resulting compressed lung volumes at peak inflation.

**FIGURE 10:**
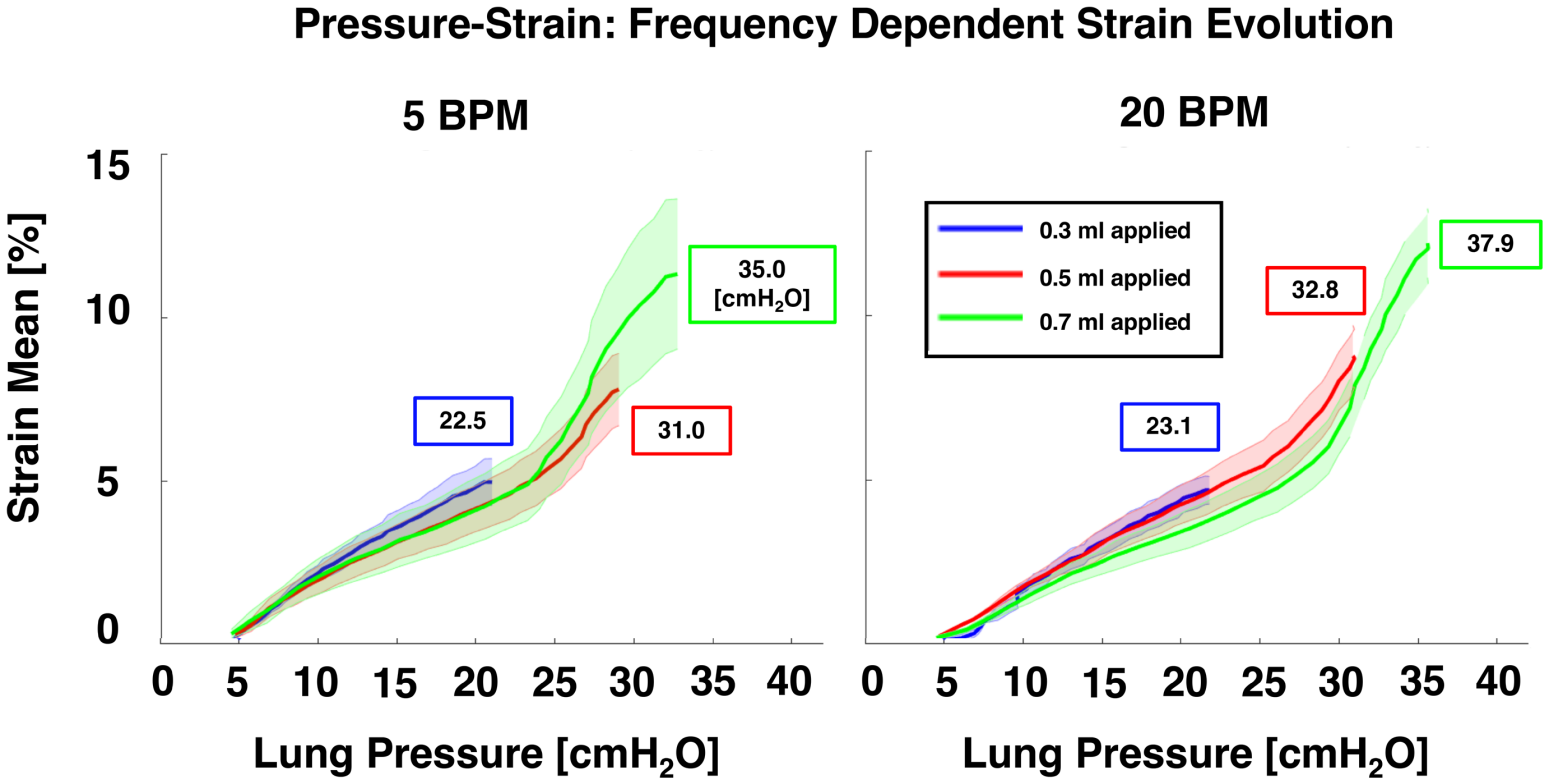
Specimens' pressure evolution of strain means for each applied inflation volume of 0.3, 0.5, and 0.7 ml and resulting peak pressure value, shown at two breathing frequencies (5 and 20 BPM). Curves represent the averaged strain mean and standard deviations across murine specimens. Show in the boxes are resulting lung pressures at end of air delivery.

The principal (major) technical strain was determined using the Trilion ARAMIS GOM software, where deformations are computed from displacement trajectories of the same particles tracked over the course of the entire deformation (Trilion Quality Systems GOM ARAMIS, 2016). The major strain behavior at peak inflation (i.e., when the maximum amount of air has been delivered from the ventilation system) was qualitatively assessed at various inflation volumes and breathing rates using topological contour maps (Figure 2). The strain distribution across the fraction of the tissue surface was quantified using histograms. The standard deviation was compared between specimens for each volume and rate, and was used to quantify dispersion of strain values relative to the mean, thus serving as measure of heterogeneity (Figure 3). At the peak inflation states, the major strain means, ranges, mode, and maximums were compared for individual mice as a function of volume and pressure (Figures 4, 5, 6, 7). Range was determined as the absolute difference between the maximum and minimum strain values, while mode was considered as the lower bound of the most populated bin of each individual specimen's strain histogram. The temporal dependency of local surface mean strains as influenced by the breathing rates over time was also evaluated (Figure 8). The local tissue strain and global lung volume evolutionary dependence was analyzed by comparing the mean strain evolution via slopes at different inflation applied volumes of 0.3, 0.5, and 0.7 ml at each breathing rate (Figure 9); the compressed lung volumes, (i.e., the measured actual lung volume with air compressibility accounted for), as opposed to the applied air volume (Sattari et al., 2020), were also compared between the two breathing rates for peak inflation. Pressure–strain curves were used to characterize local tissue compliance (Figure 10) (Limjunyawong et al., 2015; Robichaud et al., 2017), defined as the slopes of the distinct bilinear regions fit to a regression line with R^2^ > 0.9 (MATLAB, MathWorks Inc.) The bilinear curves' transition point (occurring for applied volumes greater than 0.3 ml) was found as the intersection of the two linear regression slopes and was used to determine the stage of alveolar recruitment (Eskandari et al., 2019; Lynch et al., 2003).

### 2.3 Statistical analysis and data processing

Statistical analyses comparing the two breathing rates (Figure 3, Figures 8, 9, 10) were performed using a Wilcoxon matched‐pairs signed rank test for each volume. The effect of volume was analyzed for each breathing rate (Figures 4, 5, 6, 7, Figures 9, 10) using a Friedman test with Dunn's multiple comparisons, except for the compliance slopes and transition points at applied volumes of 0.5 and 0.7 ml, where a Wilcoxon test was used to compare the two volumes (Figure 10) (MATLAB; R Foundation for Statistical Computing). The nonparametric statistical tests were used in accordance with the sample size (Krzywinski & Altman, 2014). Significance levels were designated with *p < 0.05, **p < 0.01, ***p < 0.001.

Minimal data smoothening was utilized and small facet sizes were adopted to ensure unbiased strain measurement results. Data processing included outlier tagging and removal occurring from DIC system noise due to system measurement errors. While mouse lung strain values were well below 30%, these stochastic values primarily manifested as >100% and possessed distinctly inconsistent temporal strain trajectories, indicative of solitary speckle dislocation or glare from lighting. As such, outliers were easily spotted. To consistently select such points, a threshold was set to tag data points associated with strains exceeding 150% of the interquartile range (Eskandari et al., 2019). The tagged outliers were then confirmed in manual postprocessing and removed.

## 3 RESULTS

Strain values on the surface of the murine lung were found to increase with increasing volumes and tended to increase with increasing rates (Figure 2). Highest strain values were located in the central and lower regions of the left lung and inferior lobe. Nonuniform surface strains observed on the left, inferior, and superior lobes, and became more heterogeneous with increasing applied volumes. Qualitatively, the surface heterogeneity was seen to become more pronounced with increasing breathing rate for all mice specimens.

Qualitative trends observed in Figure 2 were quantified in Figure 3. The mean surface strains were found to significantly increase with greater inflation volumes; for example, at 5 BPM for 0.3, 0.5, and 0.7 ml the mean strain was 5.0%, 7.8%, and 11.3%, respectively. The fraction of surface strain distributions tended to increase in range for increasing applied volumes; for instance, at 20 BPM for 0.3, 0.5, and 0.7 ml, the strain range was 11.2%, 17.7%, and 20.0%, respectively.

The standard deviation was observed to increase with increasing applied volumes and was significantly greater for 0.7 ml compared to 0.3 ml for both rates (*p* < 0.01 and *p* < 0.05 for 5 BPM and 20 BPM, respectively). For instance, at 5 BPM for applied volumes of 0.3, 0.5, and 0.7 ml, the standard deviation was 2.2%, 3.1%, and 3.7%, respectively, and at 20 BPM, was 2.2%, 3.4%, and 3.7% (for the increasing applied volumes, respectively). The standard deviation was similar between the two rates, with increase from 5 to 20 BPM at an applied volume of 0.5 ml.

For each applied volume, no statistical difference was found between breathing rates for the mean strain, range, mode, or maximums. Notable trends were present: at the lowest applied volume (0.3 ml), 5 BPM exhibited a smaller means and ranges compared to 20 BPM. However, at larger applied volumes of 0.5 and 0.7 ml, the opposite trend occurred; strain means and ranges were larger in 20 BPM compared to 5 BPM.

Strain means were found to increase with increasing applied volume (Figure 4). At both 5 and 20 BPM, the resulting strains were found to significantly increase with an applied volume increase from 0.3 to 0.7 ml, while between applied volumes of 0.3 and 0.5 ml, as well as between 0.5 and 0.7 ml strain mean increases were not found to be significant. Also, the relationship between local mean strain and global lung pressure was consistent across all specimens: greater lung pressures resulted in greater surface stretches. However, the relationship between strain and pressure was noted to be nonlinear compared to the near‐linear relationship between strain and volume. For increasing pressures, the slower 5 BPM frequency showed individual murine specimens tended to exhibit a steeper strain rise compared to 20 BPM.

On average, the strain range increased with increasing applied volume; however, for individual specimens between 0.5 and 0.7 ml, the range was also noted to decrease (Figure 5). At both 5 and 20 BPM, the difference between strain ranges at volumes of 0.3 and 0.7 ml were found to be statistically significant, while the differences in range between applied volumes of 0.3 and 0.5 ml, as well as between 0.5 and 0.7 ml were not found to be significant. The strain range increased between the lung pressures measured at 0.3 and 0.5 ml volumes but was noted to sometimes decrease when inflating to the highest pressures.

Strain modes significantly increased for between applied volumes of 0.3 and 0.7 ml at both breathing rates, and 20 BPM trended more linearly (Figure 6). At both rates, the difference between strain modes at applied volumes of 0.3 and 0.5 ml, as well as between 0.5 and 0.7 ml were not found to be significant. Also, while all strain modes tended to increase for increasing lung pressures, some values increased sharply while others evolved less steeply. For 20 BPM, the strain mode's relationship to lung pressure is noted to be more linear compared to 5 BPM.

The strain maximums significantly increased between applied volumes of 0.3 and 0.7 ml at both breathing rates and was noted to be rather linear (Figure 7). Increases in strain maximum between applied volumes of 0.3 and 0.5 ml as well as between 0.5 and 0.7 ml were not found to be significant. The nonlinear relationship between strain maximums and lung pressure is noted to generally evolve more steeply at higher applied volumes compared to the rise between 0.3 and 0.5 ml.

Figure 8, exhibiting the start of inflation to the maximal air delivery of both respective breathing rates at an applied volume of 0.7 ml, showed that the temporal strain evolution was linear. At a breathing rate of 5 BPM, a greater spread between specimens' time‐strain curves compared to 20 BPM was noted. For 5 BPM, the average slope was 1.9%/s, and (while not significant) was less than the rate evolution of the strain for 20 BPM, which was 8.2%/s; there was a ~ 4.3 times increase in strain rate compared to the anticipated fourfold response.

Figure 9 exhibited the relationship between the local lung strain evolution and global applied lung volume. For a breathing rate of 5 BPM, the slope of each curve was 33.7, 32.3, and 29.0%/ml, for applied volumes of 0.3, 0.5, and 0.7 ml, respectively. Similarly, for 20 BPM, the slope of each curve was 35.8, 39.8, and 35.5%/ml. The local strain evolutionary relation to the global lung volume developed similarly (slopes were similar), except between applied volumes 0.3 and 0.7 ml at 5 BPM, which were found to differ (*p* < 0.5). The strain evolution slope trended greater for 20 compared to 5 BPM. Peak compressed lung volumes (associated with the end of air delivery) are shown in boxes, and tended to be greater for 5 compared to 20 BPM.

Figure 10 showed that specimens tended to reach higher pressures at 20 compared to 5 BPM. The local compliances (defined as the initial and final slopes of the mean tissue strain vs. global lung pressure) and the transition point were calculated for all applied volumes and at each rate. The slope at an applied volume of 0.3 ml was 0.27%/cmH_2_O for 5 BPM and 0.24%/cmH_2_O for 20 BPM and was not bilinear at either rate. The two greater applied volumes (0.5 and 0.7 ml) exhibited a bilinear compliance: for an applied volume of 0.5 ml and breathing rate of 5 BPM, the average slope of the initial portion of the curve was 0.22%/cmH_2_O and 0.48%/cmH_2_O for the final slope, with a transition point of 26.0 cmH_2_O. Values increased for a breathing rate of 20 BPM: the initial slope was 0.26%/cmH_2_O and 0.52%/cmH_2_O for the final slope, with a transition point of 28.8 cmH_2_O. Similarly, for an applied volume of 0.7 ml and slower breathing rate, the average initial and final slopes were 0.23%/cmH_2_O and 0.72%/cmH_2_O, respectively, with a transition point of 25.3 cmH_2_O. At a faster breathing rate of 20 BPM, the initial slope was 0.23%/cmH_2_O, and the final slope increased to 0.87%/cmH_2_O with an increased transition point of 30.2 cmH_2_O compared to the slower breathing rate.

The final compliance slopes tended to be greater for an applied volumes of 0.7 ml compared to 0.5 ml for both breathing rates. In general, volume‐dependent comparisons of the initial compliance slope, final compliance slope, and transition point did not yield significant differences for either rate.

Similarly, rate‐dependent comparisons were not found to be significant. However, final slopes trended greater for 20 compared to 5 BPM for both 0.5 and 0.7 ml applied volumes. Additionally, the transition point tended to occur at higher pressures for the faster breathing rate for both 0.5 and 0.7 ml.

## 4 DISCUSSION

In this study, the mechanics of murine lung is characterized by associating the time‐continuous local tissue deformations to classical global PV measures for the first time. Furthermore, local and global dependencies on varying applied volumes and breathing rates are explored in terms of local strain, compliance, and tissue surface strain heterogeneity. Local strain lung behavior (e.g., the maximum, ranges, etc.) at peak inflation is found to significantly depend on global loading but not rate. Conversely, the strain evolution and continuous regional response of the tissue as a function of applied volumes and measured pressures are dependent on the breathing rate. The novel application of DIC interfaced with our custom‐designed electromechanical ventilation system facilitates such real‐time continuous measures and offers the ability to quantify pulmonary tissue‐to‐organ level kinetics and kinematics for the mouse previously unexplored to date.

### 4.1 Comparing murine strains to previous studies

For a single, same type murine lung specimen, Mariano et al found maximum strain to be 21% for a rate of 0.1 ml/s (equivalent to 6 BPM) at 0.5 ml applied volume (Mariano et al., 2020); in this study, at the same inflation volume and slighted reduced breathing rate of 5 BPM, a comparable but decreased average maximum strain response is found across multiple specimens (16.6%, Figure 3). Additionally, a DVC study characterizing strain in Sprague–Dawley rats (5–6 weeks) and CD‐1 (4–6 weeks) mice lungs found an average regional strain of 80%–100% when inflated to a maximum pressure of 30 cmH_2_O, albeit with an incomparable preload (Arora et al., 2021). At similar average pressures of 35.2–38.0 cmH_2_O with an inflation volume of 0.7 ml, our mean surface strains are 11.3% (5 BPM) and 12.2% (20 BPM) (Figures 3, 4). In the absence of additional DIC studies on murine lungs, we also compare strains to other experimental modes: Birzle et al. performed uniaxial tensile tests on isolated rat lung parenchyma strips and found a resultant strain of ~90% at 2500 Pa (25.5 cmH_2_O) (Birzle et al., 2019). At similar inflation pressures of ~25cmH_2_O, our study yields mean strain values of 5% at both 5 and 20 BPM (Figure 10) with maximum regional strains noted to be as high ~17.5% (Figure 7). Nonetheless, DIC versus DVC methods, inflation of whole organ specimens versus uniaxial measures on parenchymal components (Suki et al., 2011), use of dissimilar species and age, and different specimen preloading may explain the disparate strain values observed.

### 4.2 Volume‐dependent strain behavior

Surface strains (mean, range, maximums, etc.) measured at peak‐inflation are often found to be volume dependent and unidirectionally increase with increasing volume (Figures 4, 5, 6, 7). Conversely, the evolutionary rate of change in the mean strain is not conclusively found to be dependent on varying applied inflation volumes, and ranges from ~30%–40%/ml (Figure 9). This finding, framed in a physiological context, suggests that, while the absolute value of local strain depends on the global air volumes imposed, if the air flow rate is constant, the local deformation progresses at the same degree regardless of how much air is introduced.

### 4.3 Rate‐dependent strain behavior

The time‐dependent viscoelastic nature of the lung tissue (Bayliss & Robertson, 1939; Sattari & Eskandari, 2020) is observed in the global lung volumes at peak inflation, which tend to be greater for slower breathing (5 BPM) than faster breathing (20 BPM) for applied volumes of 0.5 and 0.7 ml (Figure 9). The prolonged tissue inflation procedure and greater lung response volumes granted by slower inflation speeds results in reduced lung pressure (Figure 10), but elevated mean strains at maximum inflation for 0.5 and 0.7 ml (Figure 3). Total murine lung capacity is 1 ml (Irvin & Bates, 2003) and while at the smaller 0.3 ml volume the strain is not found to increase between 5 and 20 BPM, closer to the physiological inflation limit of 0.5 and 0.7 ml, increased rate tends to increase the average strain, emphasizing the effect of the breathing rate at higher tidal volumes. Strain evolutionary behavior is also observed to show rate‐dependent trends, where volume–strain slopes tend to be greater for the faster inflation rate (Figure 9). The elevated strain progression may arise due to regional overinflation from imbalanced gas dispersions, which is thought to occur in high‐frequency ventilation (Herrmann et al., 2018; Kaczka et al., 2015), and may also translate to the greater strains at peak inflation for the faster rate. Nonetheless, this speculation is limited given the physiological breathing rates of mice is 109–230 BPM (Travis et al., 1981), which is greater than those examined in previous studies as well as in this current study (Limjunyawong et al., 2015; Robichaud et al., 2017). However, as stated previously, such rates were used in order to permit analyses of lung elastic, rather than dynamic, response by reducing the interference of air flow resistance as one of the factors affecting the viscosity force and pressure measurements (Bayliss & Robertson, 1939; Mount, 1955).

### 4.4 Local compliance

The manifested near‐linear direct relationship between local strain and global lung volumes (Figure 9) facilitates local compliance behavioral insights from the pressure–strain curve (Figure 10). The tissue initial compliance is seen to be generally unaffected by increasing applied volumes (Figure 10) in contrast to the local final compliance, which tends to be greater for an applied volume of 0.7 compared to 0.5 ml. Similar to classical lung bilinear PV curves, we consider the initial slope of the pressure–strain curve to represent initial alveolar recruitment and the transition point to indicate the transition to marked alveolar recruitment until deflation (Hickling, 1998; Takeuchi et al., 2001). While we observe this transition at slightly greater pressures than previous studies using murine subjects, differences may be attributable to use of different mice strains (Zosky et al., 2008). Nevertheless, the initial compliance region signifies the beginning of inflation wherein marked alveolar recruitment has not yet occurred with fewer open airways, which may explain the lack of dependency on applied volumes compared to the final compliance region, which is seen to depend on applied volumes (Engel et al., 1975). The curve bilinearity observed in this study may indicate additional recruitment mechanisms, such as collateral ventilation, wherein a thinned surfactant layer at high inflation pressures allows air passage from “mother” to connected “daughter” alveoli, thus recruiting an additional set of alveoli (Namati et al., 2008; Quiros et al., 2022). Characteristic to collateral ventilation is the resultant “popping” open of the daughter alveoli. During the inflation process, we observe a sudden elevation in strain near peak inflation for applied volumes of 0.5 and 0.7 ml. This occurs in peripheral regions of the lung and can be seen in Figure 2 for the 0.7 ml applied volume. Here, elevated strain is present in lower portion of the inferior lobe, and occurs concomitantly with an observed sudden parenchymal tissue distension.

While no significant dependence is observed between the local compliance and breathing rate, final local compliance trends greater for applied volumes of 0.5 and 0.7 ml and the point of transition tends to occur at greater pressures for 20 in comparison to 5 BPM (Figure 10). The observed steepened local tissue compliance with increasing rate is likely attributable to overinflated regions (Herrmann et al., 2018; Kaczka et al., 2015) and may result in decreased elastic recoil pressure of the lung, which suggests the potential for high ventilation frequencies to influence overventilation in subsequent cycles due to insufficient recoil (Papandrinopoulou et al., 2012; Yusen, 1999); however, the connection between regional pressure–strain analyses and localized tissue damage during multiple successive ventilation cycles is underexplored here and merits further investigation to understand the full downstream effects of breathing rates.

### 4.5 Volume‐dependent strain heterogeneity

Strain heterogeneity increases with increasing applied volumes, as seen by the significantly expanding strain range and significantly increasing standard deviation from 0.3 to 0.7 ml for both breathing rates (Figures 2 and 3) and is consistent with previous murine studies (Arora et al., 2021; Mariano et al., 2020). Dominant regions of preferential strain development have been observed in porcine specimens, where the upper region of the lung exhibits the highest strains (Maghsoudi‐Ganjeh et al., 2021; Mariano et al., 2022). However, given lung lobe morphology differences, significant size discrepancies between species, and mechanical distinctions (i.e., collateral ventilation phenomena), findings from porcine lungs cannot necessarily be extended to mouse lungs. Moreover, the strains we observe in this murine study are inhomogeneously distributed over the entire lung, rather than clustered in one specific region, similar to previous mice and rat studies quantifying volumetric strains using CT and DVC (Arora et al., 2021; Hurtado et al., 2020). Nonetheless, previous studies' characterizations of internal strains utilize 3D projections onto 2D contours, with dissimilar inflation protocols, so internal and surface strain relationships can only be conjectured.

Strain distributions of the whole expanding lung may be attributable to the underlying spatial location of airways and their material properties, (Arora et al., 2017; Eskandari et al., 2019): postexperimental dissection of porcine airways found that the regions of greatest stretch correlated with the main branches of the bronchial network (Mariano et al., 2020); yet the absence of a dominant strain region in the mouse lung compared to that of the pig suggests the role of the underlying airway network may not as readily affect the tissue deformation. Nonetheless, the physical size of the murine lung restricted our postexperimental dissection to verify.

### 4.6 Rate‐dependent strain heterogeneity

Higher regions of strain are indicative of volutrauma and the overdistension of the lung at localized regions (Auten et al., 2001). At inflation volumes of 0.5 and 0.7 ml, from slow to faster breathing rates, increased strain ranges are observed, and at a volume of 0.5 ml, standard deviation increases with faster breathing (Figure 3). These behaviors indicate increased heterogeneity, and may suggest reduced homogeneous air dispersion at faster breathing rates, supporting the theory of lung mechanical behavior asynchrony (Amini & Kaczka, 2013; Herrmann et al., 2018; Otis et al., 1956). Conversely, for 0.3 ml, the mean strains and ranges are greater at the slower compared to the faster breathing rate. This inversion may be explained by the lung compliance transition point and alveolar recruitment transition occurring at higher pressures for the faster breathing rate at both 0.5 and 0.7 ml; the delayed onset of alveolar recruitment and reduced lung volume availability to accommodate the delivered air at 20 compared to 5 BPM, indicates the tissue will have more resulting local strain in certain areas (Figures 9, 10).

Findings of increased strain heterogeneity with increasing rate are in agreement with the suggestion of Kaczka et al. that high‐frequency ventilation causes an imbalance of regional overventilation and underventilation (Kaczka et al., 2015); it is also regarded that such regional imbalances yield localized stresses (Sklar et al., 2017). Consequently, ventilating the lung at fast rates and not allowing for sufficient alveolar recruitment in various regions causes strain heterogeneity and thus lung injury, since heterogeneity is considered to be a predisposition for VILI (Beitler et al., 2016; Hurtado et al., 2020). Thus, while high‐frequency oscillatory ventilation is a means of preventing VILI by avoiding alveolar collapse (Imai & Slutsky, 2005), the heterogeneous trends suggested by this study highlight another potential avenue for VILI (Herrmann et al., 2018). While statistically insignificant, the trend toward elevated strain heterogeneity at higher rates (at inflation volumes of 0.5 and 0.7 ml) found in this study supports this notion and provides a potential explanation for why high frequencies in ventilation may be injurious in the context of rate dependent strain on the lung.

## 5 LIMITATIONS

The lung contains a network of internal elements such as airways and interweaved intraparenchymal fibers (Eskandari et al., 2018; Suki & Bates, 2011) that contribute to whole organ mechanical behavior, yet DIC is limited to measurements of topological deformations (Maghsoudi‐Ganjeh et al., 2021). While specific correlations between internal and topological tissue behaviors are not directly assessed by this work, the use of murine samples has the potential for providing such correlations, as studies at the pleural surface level in mice offer representation of structures deeper within the lung, due to the mouse lung's relatively close proximity of alveoli to the pleural surface (Mitzner et al., 2008). Planned future work will explore the degree to which surface and internal strains are associated. Although we are unable to directly report the deformation of internal constituents, DIC provides insights regarding the continuous evolution of lung deformations and associates local‐to‐global mechanics in real time, which has been unexamined to date. Such insights facilitate investigating the evolutionary behavior of the lung during the breathing cycle and pulmonary viscoelasticity, which plays a role in understanding VILI's dependency on breathing frequencies. Additionally, removal of the lung from the chest cavity poses limitations: it does not allow for analysis of changes in perfusion due to volume load, which is known to alter lung compliance (Borges et al., 2020; Schulze‐Neick, 2000). Moreover, the compliance of the lung and chest wall (ribcage and musculature) are dissimilar and can be regarded separately in their contributions to compliance of the whole respiratory system, but the behavior of the two are mechanically linked (Harris, 2005; Mitzner, 2011). Therefore, while the presence of chest wall elements would possibly affect the topological deformation values obtained in this study, the relative strain behavior in response to various volume and rates is likely to persist. Future work will aim to characterize the influence of the thoracic cavity on local strains by enclosing the lung ex vivo specimen while seeking to avoid disrupting the tissue's DIC speckle pattern as the specimen deforms during each respiratory cycle.

## 6 CONCLUSION

In this study, we characterize murine lung mechanics using DIC in conjunction with our custom‐designed ventilation device, and associate real‐time continuous local tissue deformations to classical global PV measures for the first time. Examining mice lung local mechanics is of particular interest due to their wide use in human translational studies humans and ability to induce a variety of disease states to improve our understanding of lung disease (Irvin & Bates, 2003; Quiros et al., 2022). Furthermore, the effect of various applied volumes and breathing rates on regional strains, local compliance, deformation heterogeneity, and strain evolution are quantified, providing new findings regarding the interplay between local and global pulmonary mechanics.

Furthermore, positive pressure ventilation, as employed in this study and as clinically used to artificially ventilate patients, is associated with pulmonary damage (Peták et al., 2009). Future studies will similarly investigate the interplay between local and global pulmonary mechanics as driven by negative pressure ventilation, which is the physiological mode of breathing, to better understand how artificial and natural respiration differ. Additionally, exploring diseased lung mechanics, which are likely to diverge from healthy behaviors, will better provide relevant insights for assessing VILI.

## AUTHOR CONTRIBUTIONS

M.E. conceptualized and supervised the research. T.M. Nelson, K.A.M.Q., and M.E. designed the experiments. T.M. Nelson and K.A.M.Q. performed the experiments. A.U., E.C.D., and T.M. Nordgren provided the resources. T.M. Nelson, M.E., K.A.M.Q., C.A.M., and S.S. interpreted the results. T.M. Nelson and M.E. performed the analysis of the data, prepared the figures, and wrote the manuscript. All authors approved the manuscript for submission.

## FUNDING

The authors thank funding support in part from the Hellman Fellows Program to Dr. Mona Eskandari, and the University of California Riverside Dean's Biomed Collaborative Seed Grant awarded to Drs. Mona Eskandari and Tara Nordgren.
